# Supplementary material for: Gene therapy rescues cone function in an all-cone retina mouse model with the most common cone opsin C203R missense mutation
Source: PLoS One. 2026 Jun 11;21(6):e0332684. doi: 10.1371/journal.pone.0332684 (PMC13258009; doi:10.1371/journal.pone.0332684)
Supplement: S1 Fig — (PDF) [file pone.0332684.s002.pdf]

Fig. S1

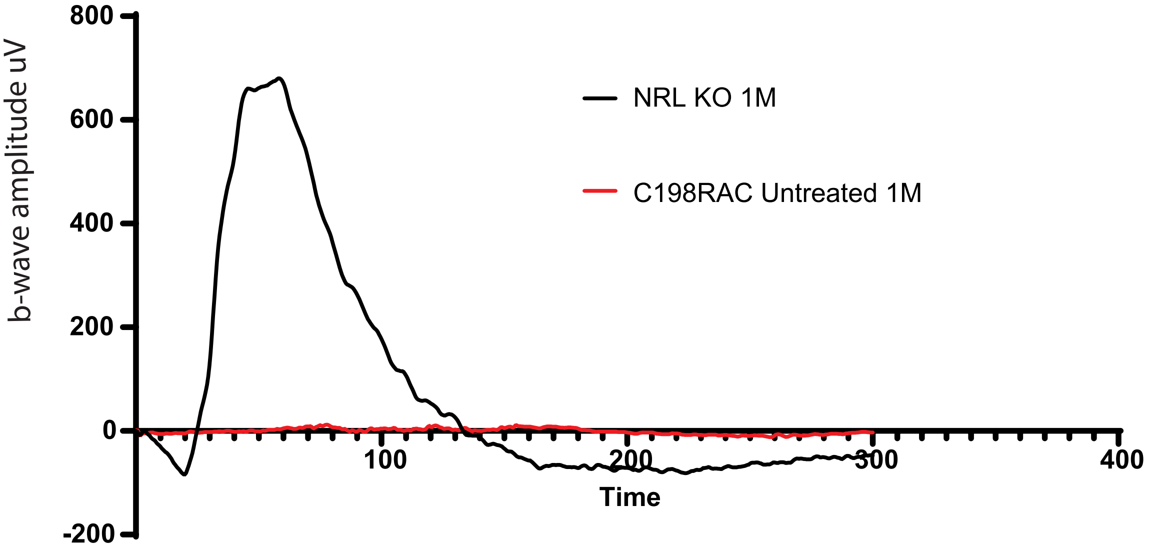


Fig. S1. Raw ERG waveforms to show that C198RAC mice showed no photopic ERG responses at one month of age .
