# Supplementary material for: Gene therapy rescues cone function in an all-cone retina mouse model with the most common cone opsin C203R missense mutation
Source: PLoS One. 2026 Jun 11;21(6):e0332684. doi: 10.1371/journal.pone.0332684 (PMC13258009; doi:10.1371/journal.pone.0332684)
Supplement: S1 File — Original uncropped images of Fig 6 (S2 Fig), Fig 5 (S3 Fig); and Fig 7 (S4 Fig). (PDF) [file pone.0332684.s003.pdf]

Fig. S2

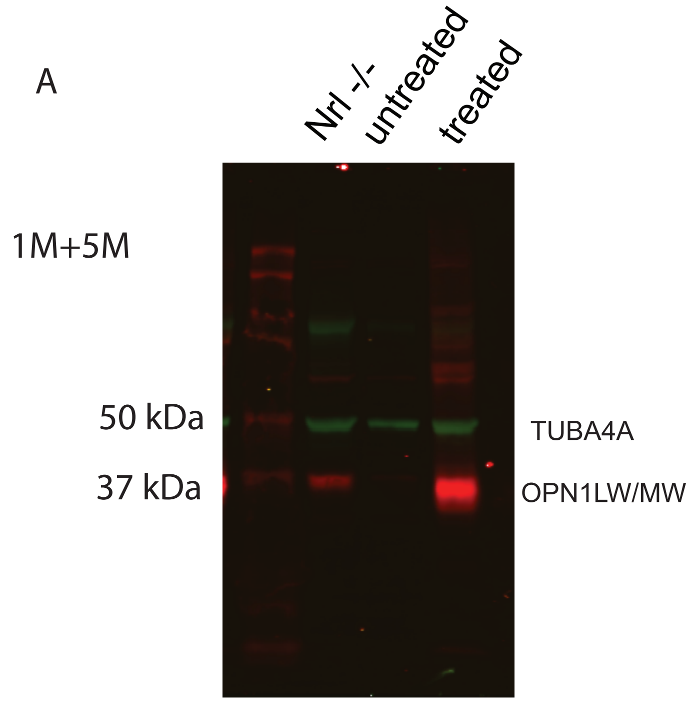

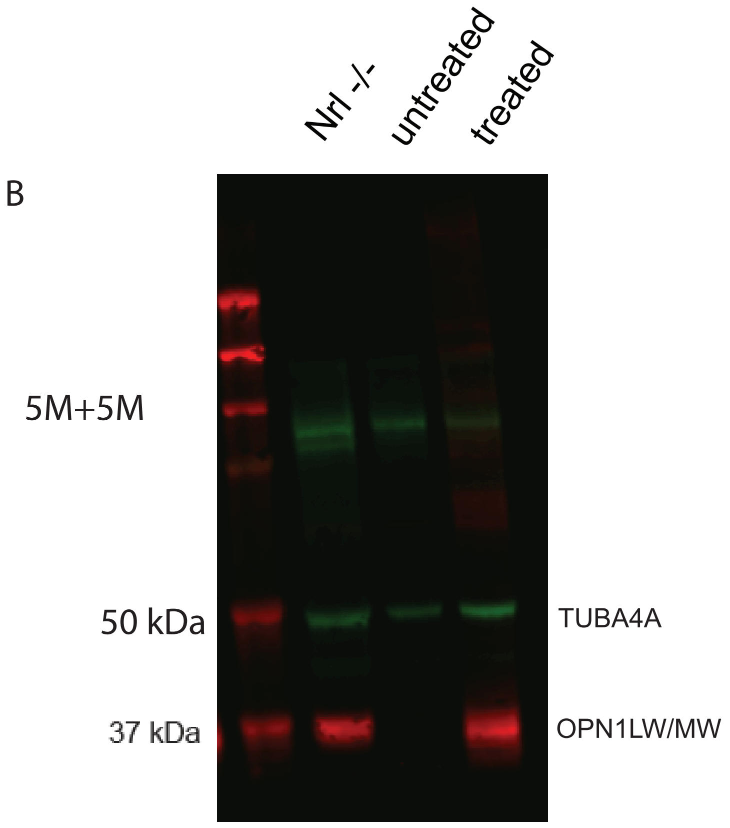

Fig. S2. uncropped western blot images of Fig 6A and 6B, A and B corresponse to Fig 6A and 6B

Fig. S3

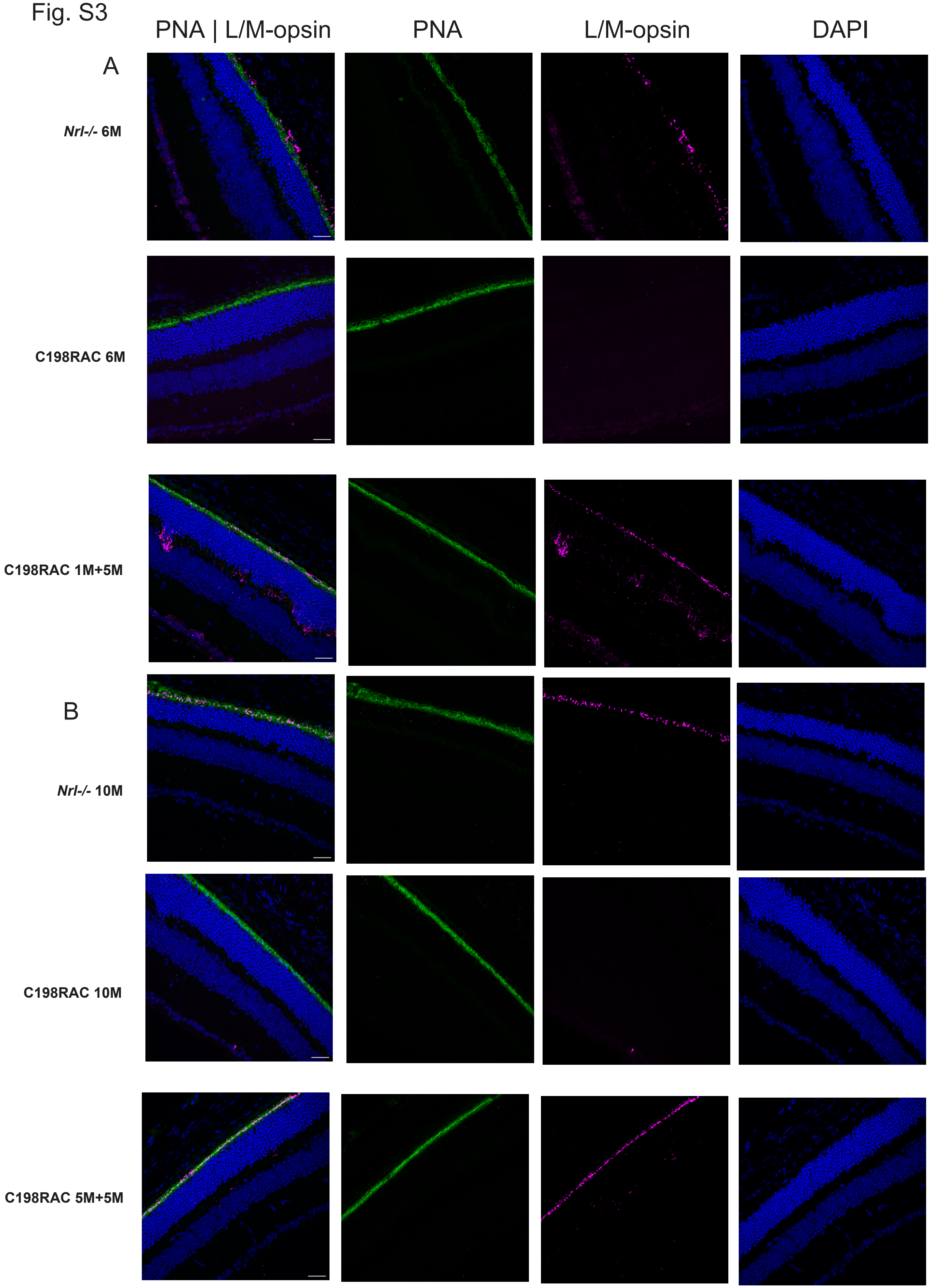

Fig. S3. uncropped images of Fig 5A and 5B. corresponding to A and B here. Individual panel channels are labled on top

Fig. S4

untreated

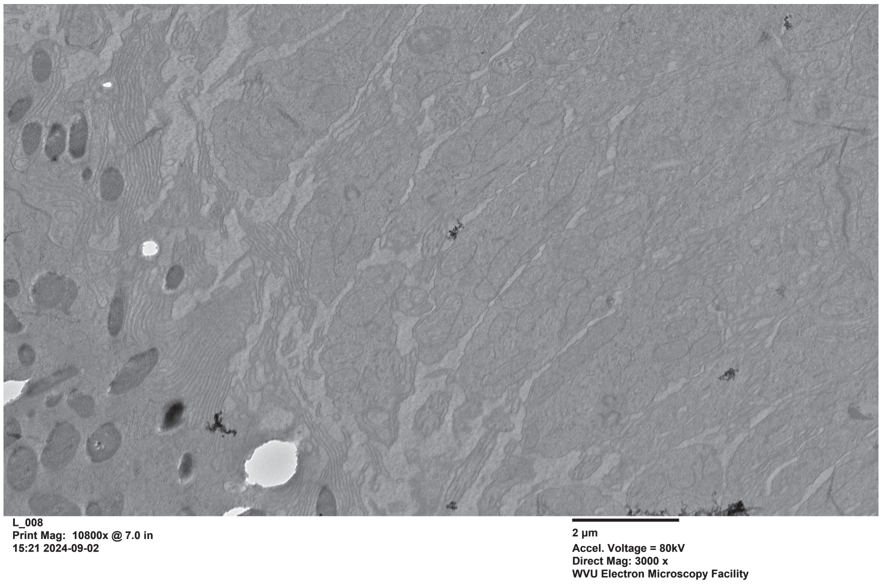

Treated

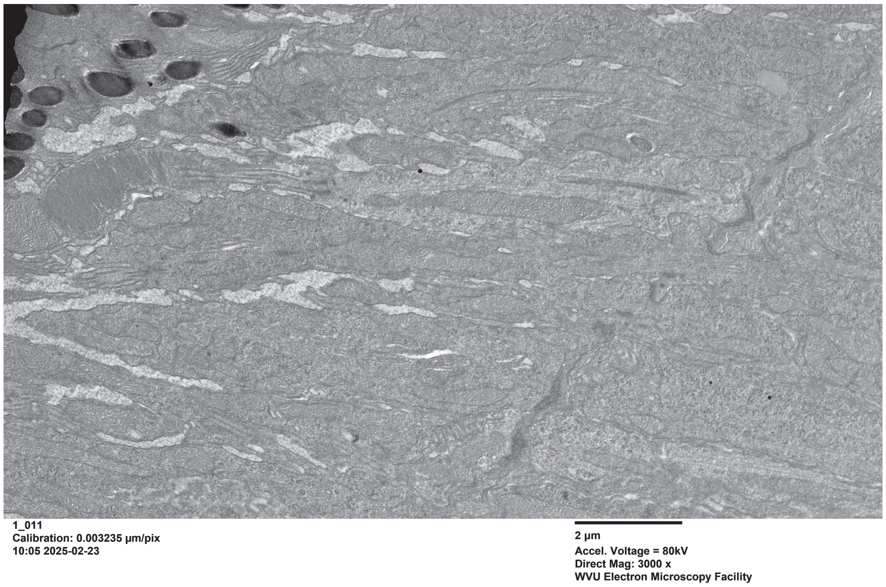

Nrl-/-

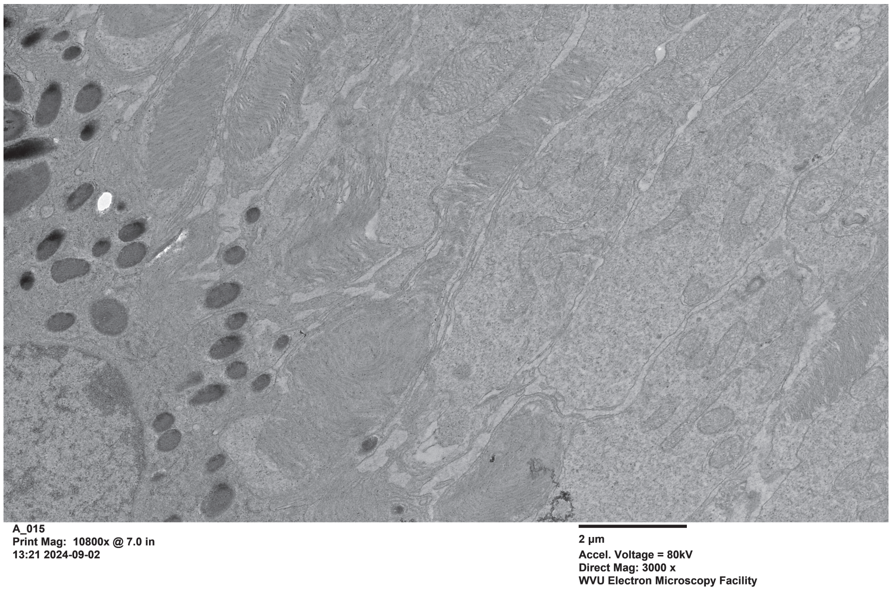

Fig. S4 uncropped images of Fig 7.
