# Supplementary figures and images for: Gene therapy rescues cone function in an all-cone retina mouse model with the most common cone opsin C203R missense mutation
Source: PLoS One. 2026 Jun 11;21(6):e0332684. doi: 10.1371/journal.pone.0332684 (PMC13258009; doi:10.1371/journal.pone.0332684)

Fig. S4

untreated

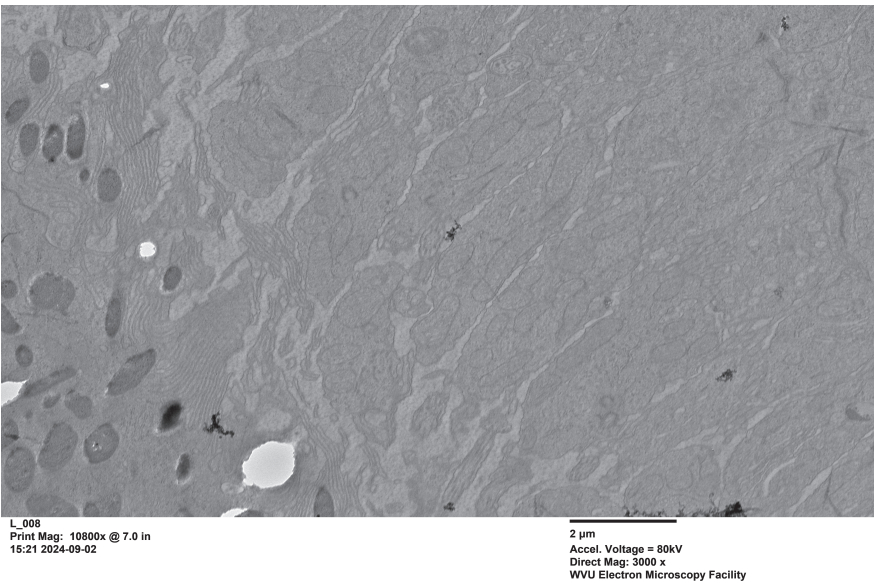

Treated

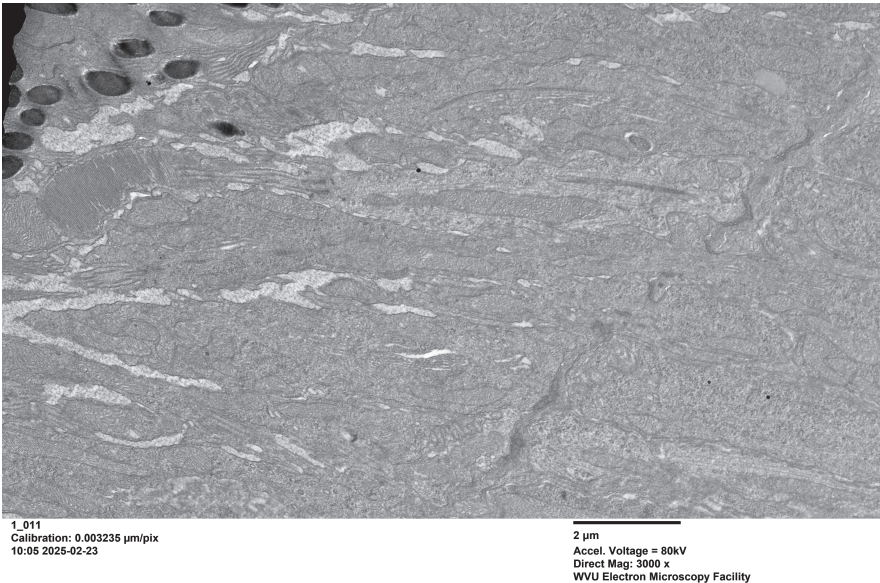

Nrl-/-

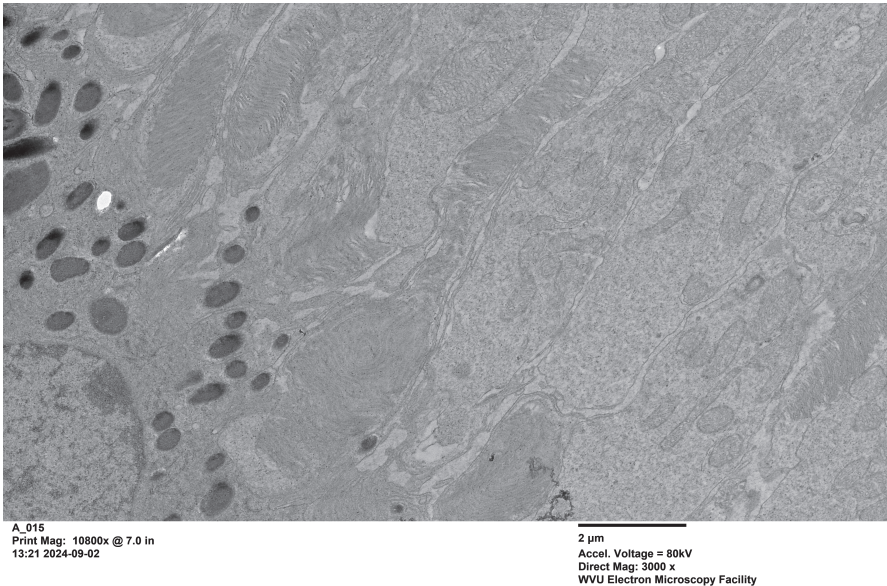

Supplement: S4 Fig — (PDF) [file pone.0332684.s006.pdf]
